# Association of Neutrophil-to-Lymphocyte Ratio and C-Reactive Protein-to-Albumin Ratio with Renal Anemia in Maintenance Hemodialysis Patients

**DOI:** 10.12669/pjms.42.7.16176

**Published:** 2026-07

**Authors:** Xiaojing Yan, Meixuan Lv, Yanli Li, Yu Sun

**Affiliations:** 1 Xiaojing Yan Department of Laboratory, First Affiliated Hospital of Qiqihar Medical College, Qiqihar, Heilongjiang Province 161041, P.R. China; 2 Meixuan Lv Department of Hemodialysis Room, First Affiliated Hospital of Qiqihar Medical College, Qiqihar, Heilongjiang Province 161041, P.R. China; 3 Yanli Li Department of Hemodialysis Room, First Affiliated Hospital of Qiqihar Medical College, Qiqihar, Heilongjiang Province 161041, P.R. China; 4 Yu Sun Department of Hemodialysis Room, First Affiliated Hospital of Qiqihar Medical College, Qiqihar, Heilongjiang Province 161041, P.R. China

**Keywords:** C-reactive protein-to-albumin ratio, Correlation, discriminative ability, Maintenance hemodialysis, Neutrophil-to-lymphocyte ratio, Renal anemia

## Abstract

**Objectives:**

To explore the correlation of the neutrophil-to-lymphocyte ratio (NLR) and the C-reactive protein-to-albumin ratio (CRP/ALB, CAR) with renal anemia in maintenance hemodialysis (MHD) patients.

**Methodology:**

Clinical data of 275 MHD patients admitted to the Hemodialysis Center of the First Affiliated Hospital of Qiqihar Medical University from December 2022 to December 2024 were retrospectively analyzed. These patients were divided into an anemia (hemoglobin [Hb] < 110 g/L) and a non-anemia (Hb ≥ 110 g/L) group based on the presence of renal anemia. Patients in the anemia group were further stratified by anemia severity into a mild subgroup (Hb > 90 g/L), a moderate subgroup (60 g/L < Hb ≤ 90 g/L), and a severe subgroup (Hb ≤ 60 g/L). The levels of NLR and CAR were measured and compared across all groups. Receiver operating characteristic (ROC) curves were used to analyze the value of each indicator in evaluating the discriminative ability of each indicator for renal anemia status. Additionally, the relationship between NLR/CAR levels and the severity of anemia was evaluated.

**Results:**

A total of 92 patients (33.5%) were diagnosed with renal anemia. The levels of NLR and CAR in the anemia group were significantly higher than those in the non-anemia group (P < 0.05). The ROC analysis showed that the areas under the curves (AUCs) for NLR and CAR in diagnosing renal anemia in MHD patients were 0.825 and 0.894, respectively (P < 0.05). With increasing anemia severity, NLR and CAR levels increased significantly (P < 0.05).

**Conclusion:**

NLR and CAR exhibit significant discriminative ability for renal anemia in MHD patients and are correlated with the severity of the condition.

## INTRODUCTION

Renal anemia is one of the most common and highly modifiable complications in patients with end-stage renal disease (ESRD) and those receiving maintenance hemodialysis (MHD).^1^,^2^ Sustained low hemoglobin (Hb) levels can exacerbate symptom burden and increase cardiovascular risks.^3^,^4^ The KDIGO Clinical Practice Guideline for Anemia in Chronic Kidney Disease indicates that the assessment and treatment of dialysis-related anemia should simultaneously address iron metabolism, inflammatory status, and response to erythropoiesis-stimulating agents (ESAs), with an emphasis on individualized monitoring and management.^5^ Moreover, Hb is not a static parameter: distinct trajectories of Hb/hematocrit correlate with the risk of renal function decline, indirectly highlighting the importance of identifying factors that influence Hb fluctuations and persistent hypohemoglobinemia.^6^

In real-world clinical practice, failure to achieve target Hb levels or suboptimal response to ESAs remains common despite iron supplementation and ESA therapy due to the combined effects of iron metabolic disorders and chronic inflammation.^7^ Studies showed the importance of maintaining a delicate balance between correcting iron deficiency and avoiding iron overload. A study by Nunes et al.^8^ demonstrated that elevated ferritin levels are associated with hepatic and bone marrow iron deposition, while Kang et al.^9^ showed that different iron statuses correlate with survival outcomes in hemodialysis patients. Inflammation can restrict iron utilization and suppress erythropoiesis, leading to functional iron deficiency and poor treatment response.^5^ Furthermore, novel therapeutic approaches such as hypoxia-inducible factor (HIF) pathway activators have emerged to promote endogenous erythropoiesis and remodel iron metabolism,^10^ with agents like Roxadustat demonstrating efficacy in maintaining Hb levels and potentially ameliorating inflammatory status in MHD patients.^11^,^12^

However, despite these therapeutic advancements, there remains a critical clinical gap: the lack of convenient, reproducible, and quantitative indicators for rapidly stratifying patients by their risk of developing renal anemia or assessing the severity of the condition. Current monitoring often relies on lagging markers, and there is a need for bedside tools that reflect the complex interplay of inflammation and nutrition that drives persistent hypohemoglobinemia.

The neutrophil-to-lymphocyte ratio (NLR), derived from routine complete blood count (CBC), can reflect systemic inflammation and immune imbalance.^13^ Han et al.^14^ found that elevated NLR correlates with poor nutritional status in patients with chronic kidney disease, and is associated with increased risk of adverse outcomes in dialysis cohorts.^15^ The C-reactive protein-to-albumin ratio (CRP/ALB, CAR) integrates inflammatory intensity and protein nutritional reserve into a single biomarker.^16^ Recent studies have shown that CAR can serve as a nutritional biomarker for the malnutrition-inflammation complex syndrome (MICS) in the MHD population^16^ and is independently associated with the Malnutrition-Inflammation Score (MIS).^17^ Nevertheless, the associations of NLR and CAR with renal anemia in MHD patients, as well as their value in identifying anemia risk and stratifying anemia severity, remain unclear.

This study retrospectively enrolled MHD patients to explore the correlations of NLR and CAR with renal anemia, and to evaluate their discriminative performance and relationship with anemia severity. The study may provide novel clinical insights for the early identification and individualized management of dialysis-related anemia.

## METHODOLOGY

Clinical data of 275 MHD patients admitted to the hemodialysis center of the First Affiliated Hospital of Qiqihar Medical College from December 2022 to December 2024 were assessed.

### Ethical approval:

The ethics committee of our hospital approved this study with the number 2024-019-01; Date: May 8, 2024.

### Inclusion criteria:

- Meets diagnostic criteria of CRF in the guidelines for the diagnosis and treatment of chronic renal failure.
- Stable clinical status and baseline blood system function (excluding anemia-related parameters).
- MHD > 3 months.
- Age range from 18 to 75 years.
- Complete clinical data.

### Exclusion criteria:

- Acute coronary syndrome.
- Complicated with severe infection or malignant tumor.
- Complicated with severe cardiovascular and cerebrovascular diseases.
- A history of blood system diseases.
- Patients with gastrointestinal bleeding or other acute blood loss within half a year, and a history of blood transfusion within three months.
- Known primary immunodeficiency or active autoimmune diseases (such as systemic lupus erythematosus, vasculitis).
- Current or recent (within 6 months) use of immunosuppressive agents or corticosteroids.

### According to the 2012 Kidney Disease:

Improving Global Outcomes (KDIGO) guidelines, the recommended target Hb level for MHD patients is 110–120 g/L. Therefore, in this study, patients with Hb < 110 g/L were assigned to the anemia group (n=92), and those with Hb ≥ 110 g/L were assigned to the non-anemia group (n=183). Patients in the anemia group were further subdivided by anemia severity into a mild anemia subgroup (Hb > 90 g/L), a moderate anemia subgroup (60 g/L < Hb ≤ 90 g/L), and a severe anemia subgroup (Hb ≤ 60 g/L).

### Data Collection:

Baseline Data: General clinical information, including age, gender, height, body weight, dialysis vintage, and primary disease.

### Laboratory Parameters:

Hb levels, absolute neutrophil count, absolute lymphocyte count, albumin, and C-reactive protein levels. The neutrophil-to-lymphocyte ratio (NLR) and C-reactive protein-to-albumin ratio (CAR) were calculated using the following formulas:

NLR = Absolute Neutrophil Count / Absolute Lymphocyte Count

CAR = C-reactive Protein / Albumin

### Statistical Analysis:

All data were analyzed using SPSS 27.0 software. Normality of distribution was evaluated via the Shapiro-Wilk test. Normally distributed continuous data were expressed as mean ± standard deviation, while non-normally distributed continuous data were presented as median and interquartile range (IQR). Comparisons between two groups were performed using the independent-samples t-test or the Mann-Whitney U test, as appropriate. Categorical data were expressed as frequency (n) and percentage (%). Inter-group comparisons were performed using the Chi-square test or Fisher’s exact test, as appropriate. To minimize Type-I errors when comparing specific primary disease categories, Bonferroni correction was applied, with the adjusted significance level set at α′=0.05/6≈0.00833. The receiver operating characteristic (ROC) curve was employed to assess the discriminative ability of each indicator for renal anemia status, with the optimal cut-off values determined by maximizing the Youden index (Sensitivity + Specificity - 1). The Spearman correlation analysis was used to determine the relationship between NLR/CAR levels and anemia severity. A value of P < 0.05 was considered statistically significant.

## RESULTS

A total of 275 MHD patients (156 males and 119 females) met the study’s eligibility criteria, with ages ranging from 35 to 75 years and a mean age of 56.7±8.5 years. Among them, 92 patients (33.5%) were diagnosed with renal anemia, including 23 cases of mild, 40 cases of moderate, and 29 cases of severe anemia. There were no statistically significant differences in the baseline characteristics between the anemia group and the non-anemia group (p>0.05; Table-I). NLR and CAR levels in the anemia group were significantly higher than those in the non-anemia group (p<0.05; Table-II).

**Table-I:** Baseline characteristics.

Characteristics	Anemia group (n=92)	Non-anemia group (n=183)	χ^2^/t/Z	p
Sex, n(%)			0.526	0.468
Male	55 (59.8)	101 (55.2)		
Female	37 (40.2)	82 (44.8)		
Age (years)	57.8±8.9	56.2±8.2	1.412	0.159
BMI (kg/m²)	24.2±2.4	24.0±2.4	0.605	0.545
Dialysis Vintage (months)	48 (34-65)	45 (35-58)	0.830	0.407
***Primary Disease, n(%)***				
Chronic Nephritis Syndrome	41 (44.6)	88 (48.1)	0.305	0.581
Diabetic Nephropathy	24 (26.1)	38 (20.8)	0.993	0.319
Hypertensive Renal Damage	15 (16.3)	25 (13.7)	0.344	0.558
Nephrotic Syndrome	5 (5.4)	17 (9.3)	1.236	0.266
Polycystic Kidney Disease	4 (4.3)	11 (6.0)	0.328	0.567
Others	3 (3.3)	4 (2.2)		0.690
***Anemia Severity, n(%)***				
Mild	23 (25.0)			
Moderate	40 (43.5)			
Severe	29 (31.5)			

***Note:*** Data are presented as mean ± SD, n (%), or median (IQR). Continuous variables were compared using Student's t-test or Mann-Whitney U test, as appropriate. Categorical variables were compared using the χ^2^ test or Fisher's exact test. For comparisons of individual primary disease categories, Bonferroni correction was applied (α'=0.00833). Anemia severity was assessed only within the anemia group.

**Table-II:** NLR and CAR levels.

Variables	Anemia group (n=92)	Non-anemia group (n=183)	p
NLR	5.01 (3.45-6.89)	2.67 (1.79-3.58)	<0.001
CAR	0.30 (0.25-0.38)	0.16 (0.12-0.22)	<0.001

**Fig.1:**
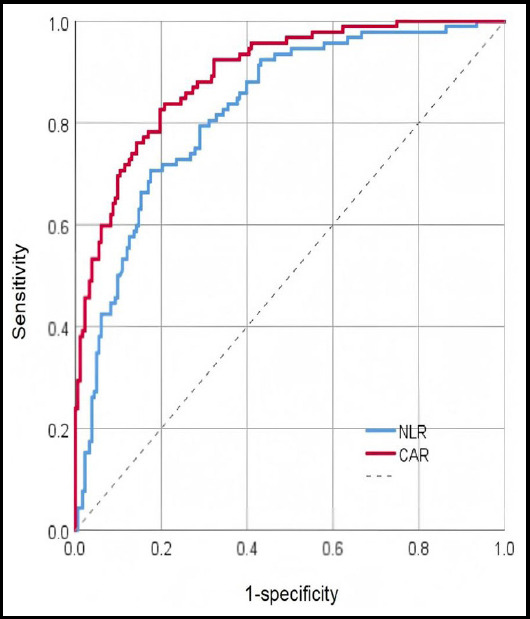
ROC curve of NLR and CAR in the diagnosis of renal anemia.

As shown in Fig.1 and summarized in Table-III, ROC analysis showed that the area under the curve (AUC) for NLR and CAR in diagnosing renal anemia in MHD patients was 0.825 and 0.894, respectively (p<0.05). With increasing anemia severity, NLR and CAR levels also increased significantly (p<0.05; Table-IV). Spearman correlation analysis showed that NLR and CAR levels were positively correlated with the degree of anemia (p<0.05; Table-V).

**Table-III:** Efficacy analysis of NLR and CAR in discriminating renal anemia status.

Variables	AUC	Sensitivity (%)	Specificity (%)	Cutoff value	AUC 95%CI	p
NLR	0.825	70.7	82.5	3.98	0.774-0.876	<0.001
CAR	0.894	82.6	80.6	0.23	0.856-0.932	<0.001

**Table-IV:** Comparison of NLR and CAR levels in patients with different degrees of anemia.

Variables	Mild group (n=23)	Moderate group (n=40)	Severe group (n=29)	F	p
NLR	3.79±1.46	5.00±1.82	7.27±2.51	21.057	<0.001
CAR	0.25±0.07	0.31±0.08	0.43±0.13	24.224	<0.001

**Table-V:** Correlation Analysis between NLR and CAR levels and anemia degree.

	NLR		CAR	
	r	P	r	p
Anemia degree	0.554	<0.001	0.572	<0.001

## DISCUSSION

A retrospective analysis of 275 patients undergoing MHD demonstrated that both NLR and CAR were significantly elevated in the anemia group (Hb < 110 g/L) compared with the non-anemia group, and their levels increased progressively with worsening anemia severity. Both NLR and CAR showed good discriminative ability for identifying anemia, with AUCs of 0.825 and 0.894, respectively; CAR had the higher AUC. These results suggest that routinely obtainable inflammation-nutrition related ratio indicators may be closely associated with the occurrence and severity of renal anemia in MHD patients. The significantly elevated NLR and CAR in the anemia group reflect a systemic state of micro-inflammation and nutritional depletion.^13^,^14^ Neutrophilia is a hallmark of the persistent micro-inflammatory environment in MHD patients, whereas lymphopenia is typically associated with protein-energy wasting and immune dysfunction.^14^,^15^ Similarly, CAR, as a composite of CRP (an acute-phase protein) and albumin (a negative acute-phase protein), serves as a robust indicator of the Malnutrition-Inflammation Complex Syndrome (MICS).^16^,^17^ The synergistic elevation of these markers suggests that patients with severe anemia are often trapped in a state where inflammation and malnutrition exacerbate one another.^16^,^18^,^19^

Previous studies on inflammatory and nutritional indicators in the dialysis population have mostly taken mortality or cardiovascular events as endpoints. Prospective studies have indicated that nutritional status, uremic toxins, and metabolic inflammatory markers can predict the risk of cardiovascular mortality in dialysis patients.^18^ A multicenter cohort study by Chen et al. has also supported the use of inflammatory and nutritional indices for stratifying overall survival.^19^ Zhu et al.^20^ proposed that the neutrophil-to-platelet ratio (NPAR) is independently associated with mortality in hemodialysis patients, and a similar conclusion has been reached in the peritoneal dialysis population.^21^ Wang et al.^22^ reported that the combination of the geriatric nutritional risk index (GNRI) with NLR, as well as the prognostic nutritional index (PNI) with NLR, can improve the mortality predictive ability^23^ while the combination of NLR with other leukocyte ratios can also be applied for risk stratification.^24^ In addition, studies demonstrated the prognostic significance of NLR and its interaction with vascular access-related factors, and the value of multi-index integration was further supported by multivariate nomogram models.^25^,^26^ Compared with the previous research, this study shifted the endpoint to the modifiable anemia phenotype, suggesting that these indicators may reflect the upstream background of persistently low Hb levels.

The biological mechanism linking high NLR/CAR to renal anemia centers on the “inflammation-hepcidin-erythropoiesis” axis.^5^,^7^ Pro-inflammatory cytokines (such as IL-6 and TNF-α) associated with high NLR and CAR levels stimulate the liver to overproduce hepcidin, the master regulator of iron homeostasis.^7^,^27^ Excessive hepcidin degrades ferroportin, sequestering iron within macrophages and hepatocytes and leading to “functional iron deficiency,” which starves developing erythrocytes of the iron required for hemoglobin synthesis.^7^ Furthermore, these cytokines directly suppress the proliferation of erythroid progenitor cells in the bone marrow and attenuate the response to erythropoiesis-stimulating agents (ESAs), causing marked EPO resistance.^12^ This cascade provides a compelling explanation for why chronic inflammation, as captured by elevated NLR and CAR, correlates so strongly with the aggravation of renal anemia.^28^ Ribeiro et al.^27^ reported that hemodialysis can increase NLRP3 inflammasome expression and exacerbate oxidative stress. At the level of clinical events, NLR and CAR have been used for identifying infection risks; for instance, studies on the prediction of catheter-related bloodstream infections have indicated that both indicators have a certain predictive value,^28^ and that the CRP/prealbumin ratio under infectious stress is also associated with the prognosis of hemodialysis patients.^29^ A systematic analysis of a large MHD cohort by Thijssen et al.^30^ showed that, in CHD patients, serum albumin levels in CAR are determined by a complex interplay of inflammation, nutrition, and dialysis efficacy. The association between CAR and long-term dysfunction of arteriovenous fistulas also suggests that vascular access-related inflammation may affect the interpretation of this indicator.^31^

In addition to the inflammation-nutrition axis, the complex phenotype of dialysis-related anemia may be shaped by multiple-pathway factors. Han et al.^14^ reported that elevated NLR was associated with poor nutritional status in patients with CKD. Chiang et al.^32^ further demonstrated that lean body mass depletion is associated with EPO resistance in patients receiving ESAs. In addition, Hamano et al.^33^ showed that fibroblast growth factor 23 (FGF23), endogenous EPO levels, and ESA dosage are associated with EPO resistance. The observed correlations between these parameters and Hb levels suggest that the mineral–bone metabolism axis may play an important role in regulating anemia.^34^ Although this study did not directly assess ESA resistance, the inflammation-nutrition phenotype, as reflected by NLR and CAR, may interact synergistically with these mechanistic pathways, thereby exhibiting a stable association with Hb levels and anemia severity.

The behavioral and psychological status of dialysis patients may indirectly affect nutritional and anemia phenotypes through dietary intake and inflammatory responses.^35^ Numerous studies focusing on nutritional knowledge, attitudes, and practices have identified distinguishable behavioral profiles among patients.^36^ Moreover, correlations between biochemical parameters and depression, anxiety, and stress^37^ further highlight the importance of comprehensive management in clinical settings.

### Strength of this study:

It selected renal anemia, a highly prevalent complication with well-defined clinical intervention potential in the dialysis population, as the primary endpoint, and conducted quantitative assessments based on routinely obtainable NLR and CAR, providing discriminative information applicable for clinical preliminary screening and stratified management. Additionally, this study extends the evidence linking inflammation–nutrition indicators, previously applied primarily for survival prognostic stratification, to the anemia phenotype. Therefore, the study provides more actionable insights for individualized anemia management.

### Limitations

First, the retrospective nature of this study limits its ability to establish causal inference; furthermore, no dynamic modeling was performed to track the longitudinal variations of NLR/CAR and Hb levels over time. Second, the lack of multivariable logistic regression analysis means that the independent association between these markers and renal anemia remains to be definitively established, as we could not fully adjust for residual confounding factors such as occult infections, dialysis adequacy, and volume status. Third, the failure to systematically evaluate variables related to EPO resistance and mineral-bone metabolism restricts our capacity for a deep mechanistic interpretation of the findings. Finally, the optimal cut-off values for NLR and CAR were derived from a single-center population without internal or external validation; consequently, their generalizability and clinical applicability should be interpreted with caution until confirmed by independent, larger-scale cohorts.

## CONCLUSION

This study demonstrated that in MHD patients, elevated NLR and CAR are closely associated with the onset and aggravated severity of renal anemia, and both indicators exhibit good discriminative ability for anemia. Among them, CAR has superior discriminative efficacy over NLR. As composite inflammation-nutrition indicators derived from routine complete blood count and biochemical tests, NLR and CAR can provide convenient and feasible references for the early identification and risk stratification of renal anemia in dialysis patients. Nevertheless, multicenter prospective studies are still needed to further validate their threshold values and clinical utility.

### Recommendations

Looking forward, several key areas warrant further investigation to build upon our findings. First, large-scale, multi-center prospective studies are essential to transition from identifying correlations toward establishing causality, further validating the predictive value of NLR and CAR in the onset and progression of renal anemia. Second, future research should prioritize the longitudinal and dynamic monitoring of these markers to explore whether their fluctuations can serve as sensitive indicators for adjusting ESA dosages or early identification of ESA resistance. Finally, interventional translational studies are required to evaluate whether targeted anti-inflammatory and nutritional strategies aimed at lowering NLR and CAR levels can directly improve anemia outcomes and reduce ESA requirements in the MHD population.

## References

[1] Khan A, Ghulam Hussain S, Mushtaq S, Abbas S, Dong Y, Feng W (2024). Prevalence and management of anemia and impact of treatment burden on health-related quality of life in chronic kidney disease and dialysis patients. J Pharm Policy Pract.

[2] Yin S, Du Y, Guo Y, Guo G, Sun D, Yao L (2022). Multifactorial analysis of renal anemia-associated substandard hemoglobin levels and prevalence of anemia in patients on maintenance hemodialysis in Liaoning Province: a cross-sectional study. Ann Palliat Med.

[3] Marques Vidas M, Portolés J, Cobo M, Gorriz JL, Nuñez J, Cases A (2025). Anemia Management in the Cardiorenal Patient: A Nephrological Perspective. J Am Heart Assoc.

[4] Kohara C, Yamada S, Tanaka S, Hiyamuta H, Kitamura H, Arase H (2024). Blood Hemoglobin Concentrations and the Incidence of Lower Extremity Peripheral Arterial Disease in Patients Undergoing Hemodialysis: 10-Year Outcomes of the Q-Cohort Study. J Am Heart Assoc.

[5] Kidney Disease: Improving Global Outcomes (KDIGO) Anemia Work Group (2026). KDIGO 2026 Clinical Practice Guideline for the Management of Anemia in Chronic Kidney Disease (CKD). Kidney Int.

[6] Fu LZ, Chen HF, Shen YH, Zhang XL, Tang F, Hu XX (2025). Time-updated patterns of hemoglobin and hematocrit and the risk of CKD progression. Front Endocrinol (Lausanne).

[7] Begum S, Latunde-Dada GO (2019). Anemia of Inflammation with An Emphasis on Chronic Kidney Disease. Nutrients.

[8] Nunes LLA, Dos Reis LM, Osorio R, Guapyassú HKA, Moysés RMA, Leão Filho H (2024). High ferritin is associated with liver and bone marrow iron accumulation: Effects of 1-year deferoxamine treatment in hemodialysis-associated iron overload. PLoS One.

[9] Kang SH, Kim BY, Son EJ, Kim GO, Do JY (2023). Association between Iron Status and Survival in Patients on Chronic Hemodialysis. Nutrients.

[10] Haase VH, Tanaka T, Koury MJ (2024). Hypoxia-inducible factor activators: a novel class of oral drugs for the treatment of anemia of chronic kidney disease. Hematology Am Soc Hematol Educ Program.

[11] Akizawa T, Iwasaki M, Yamaguchi Y, Majikawa Y, Reusch M (2020). Phase 3, Randomized, Double-Blind, Active-Comparator (Darbepoetin Alfa) Study of Oral Roxadustat in CKD Patients with Anemia on Hemodialysis in Japan. J Am Soc Nephrol.

[12] Zhao XN, Liu SX, Wang ZZ, Zhang S, You LL (2023). Roxadustat alleviates the inflammatory status in patients receiving maintenance hemodialysis with erythropoiesis-stimulating agent resistance by increasing the short-chain fatty acids producing gut bacteria. Eur J Med Res.

[13] Buonacera A, Stancanelli B, Colaci M, Malatino L (2022). Neutrophil to Lymphocyte Ratio: An Emerging Marker of the Relationships between the Immune System and Diseases. Int J Mol Sci.

[14] Han Q, Lin S, He F, Zhang R, Xie X, Qing F (2022). A high neutrophil to lymphocyte ratio is associated with poor nutritional status in chronic kidney disease patients. Br J Nutr.

[15] Carollo C, Mancia E, Sorce A, Altieri C, Altieri D, Brunori G (2025). Prognostic impact of neutrophil-to-lymphocyte ratio and vascular access in patients on chronic hemodialysis. J Nephrol.

[16] Li M, Jiang Z, Zhang D (2025). The C-reactive protein/albumin ratio as a nutritional biomarker in maintenance hemodialysis patients: a cross-sectional study of malnutrition-inflammation status assessment. Front Med (Lausanne).

[17] Tur K, Güçlü A (2024). Independent Association Between Malnutrition Inflammation Score and C Reactive Protein/Albumin Ratio in Hemodialysis Patients. J Inflamm Res.

[18] Czaja-Stolc S, Potrykus M, Ruszkowski J, Dębska-Ślizień A, Małgorzewicz S (2025). Nutritional Status, Uremic Toxins, and Metabo-Inflammatory Biomarkers as Predictors of Two-Year Cardiovascular Mortality in Dialysis Patients: A Prospective Study. Nutrients.

[19] Chen X, Wang G, Yin X, Liu W, Huang H, Li D (2025). Inflammatory and nutritional indices for overall survival in Hemodialysis patients: a multicenter cohort study. BMC Nephrol.

[20] Zhu J, Shi R, Li X, Liu M, Yu L, Bai Y (2025). Association between neutrophil percentage-to-albumin ratio and mortality in Hemodialysis patients: insights from a prospective cohort study. BMC Nephrol.

[21] Arshad RG, Toori KU (2024). Correlation between neutrophil to lymphocyte ratio and C-reactive protein in diverse disease states in hospitalized patients. Pak J Med Sci.

[22] Wang J, Huang LJ, Li B, Xu MC, Yang L, Deng X (2023). Combined evaluation of Geriatric nutritional risk index and Neutrophil to lymphocyte ratio for predicting all-cause and cardiovascular mortality in hemodialysis patients. PLoS One.

[23] Xu F, Cheng S, Shu P, Liang Y, Wang X, Bai H (2025). Combined predictive value of prognostic nutritional index and neutrophil to lymphocyte ratio for all-cause mortality risk in maintenance hemodialysis patients: a cohort study followed for 5 years. BMC Nephrol.

[24] Liao J, Wei D, Sun C, Yang Y, Wei Y, Liu X (2022). Prognostic value of the combination of neutrophil-to-lymphocyte ratio, monocyte-to-lymphocyte ratio and platelet-to-lymphocyte ratio on mortality in patients on maintenance hemodialysis. BMC Nephrol.

[25] Cakmak U, Sevimli N, Akkaya S, Merhametsiz O (2025). Prediction of Mortality in Hemodialysis Patients Using Inflammation- and Nutrition-Based Indices. J Pers Med.

[26] Sun L, Zhang Y, Zuo X, Liu Y (2024). A novel nomogram for predicting mortality risk in young and middle-aged patients undergoing maintenance hemodialysis: a retrospective study. Front Med (Lausanne).

[27] Ribeiro M, Cardozo LFMF, Coutinho-Wolino KS, Ribeiro-Alves M, Mafra D (2025). Hemodialysis Intensifies NLRP3 Inflammasome Expression and Oxidative Stress in Patients with Chronic Kidney Disease. Int J Mol Sci.

[28] Zhao Z, Yuan S, Zhang X, Li H, Liu X, Zhang L (2025). Neutrophil-to-lymphocyte ratio and CRP-to-albumin ratio in the prediction of catheter-related bloodstream infection among maintenance hemodialysis patients: a synergistical optimization algorithm. Front Med (Lausanne).

[29] Bayrak M (2019). Predictive value of C-Reactive Protein/Albumin ratio in patients with chronic complicated diabetes mellitus. Pak J Med Sci.

[30] Thijssen S, Wystrychowski G, Usvyat L, Kotanko P, Levin NW (2007). Determinants of serum albumin concentration analyzed in a large cohort of patients on maintenance hemodialysis. J Ren Nutr.

[31] Hu S, Wang R, Ma T, Lei Q, Yuan F, Zhang Y (2023). Association between preoperative C-reactive protein to albumin ratio and late arteriovenous fistula dysfunction in hemodialysis patients: a cohort study. Sci Rep.

[32] Chiang WF, Hsiao PJ, Wu KL, Chen HM, Chu CM, Chan JS (2022). Investigation of the Relationship between Lean Muscle Mass and Erythropoietin Resistance in Maintenance Haemodialysis Patients: A Cross-Sectional Study. Int J Environ Res Public Health.

[33] Hamano N, Komaba H, Tanaka H, Takahashi H, Takahashi Y, Hyodo T (2025). Fibroblast Growth Factor 23, Endogenous Erythropoietin, Erythropoiesis-Stimulating Agents, and Erythropoietin Resistance in Hemodialysis Patients. Am J Nephrol.

[34] Liu QF, Sun ZY, Tang XF, Yu LX, Li SS (2025). Relationship of sKlotho with hemoglobin level in patients undergoing maintenance hemodialysis: a case-control study. Ther Adv Chronic Dis.

[35] Wang LJ, Wu MS, Hsu HJ, Wu IW, Sun CY, Chou CC (2012). The relationship between psychological factors, inflammation, and nutrition in patients with chronic renal failure undergoing hemodialysis. Int J Psychiatry Med.

[36] Xu Y, Chen Z, Tang X, Xia X, Zhao N, Zou S (2025). Latent profile analysis of nutrition knowledge, attitudes, and practices and their influencing factors in maintenance hemodialysis patients. Sci Rep.

[37] Zaragoza Fernández GM, Jiménez Mayor E, Chandu Nanwani A, Rodríguez Tudero C, De La Flor JC, Fernández Castillo R (2025). Biochemical Associations with Depression, Anxiety, and Stress in Hemodialysis: The Role of Albumin, Calcium, and β2-Microglobulin According to Gender. Biomedicines.

